# Editorial: The Cytoskeleton and Cellular Compartmentation: Cilia as Specialized Cellular Domains

**DOI:** 10.3389/fcell.2021.777758

**Published:** 2021-10-22

**Authors:** Francesc R. Garcia-Gonzalo, Helena Soares, Susana S. Lopes, Takanari Inoue

**Affiliations:** ^1^Departamento de Bioquímica, Facultad de Medicina, Universidad Autónoma de Madrid (UAM), Madrid, Spain; ^2^Instituto de Investigaciones Biomédicas “Alberto Sols”, Consejo Superior de Investigaciones Científicas (CSIC)-UAM, Madrid, Spain; ^3^Instituto de Investigación del Hospital Universitario de La Paz (IdiPAZ), Madrid, Spain; ^4^CIBER de Enfermedades Raras (CIBERER), Instituto de Salud Carlos III (ISCIII), Madrid, Spain; ^5^Escola Superior de Tecnologia da Saúde de Lisboa, Instituto Politécnico de Lisboa, Lisbon, Portugal; ^6^Centro de Química Estrutural - Faculdade de Ciências da Universidade de Lisboa, Lisbon, Portugal; ^7^CEDOC, Chronic Diseases Research Center, Nova Medical School/Faculdade de Ciências Médicas, Universidade Nova de Lisboa, Lisbon, Portugal; ^8^Department of Cell Biology, School of Medicine, Johns Hopkins University, Baltimore, MD, United States; ^9^Center for Cell Dynamics, School of Medicine, Johns Hopkins University, Baltimore, MD, United States

**Keywords:** primary cilia, ciliopathies, ciliogenesis, motile/immotile cilia, cilia signaling

## Introduction

Cilia are fascinating microtubule-based compartments that project from the cell surface. Cilia can be functionally classified as motile (generating cell movement or fluid flow) or immotile (primary cilia, which transduce mechanical, optical, or chemical signals through a variety of pathways, such as Hedgehog, GPCR, Notch, Wnt, Hippo, mTOR, PDGFR, or TGFβ; Anvarian et al., 2019). Structurally, cilia are composed of a microtubule shaft (axoneme) that elongates from a membrane-anchored centriole (basal body) and are covered by a highly specialized ciliary membrane. Most human cell types form cilia, dysfunction of which leads to diseases such as cancer and ciliopathies, a diverse group of disorders affecting eyes, kidneys and brain, among other organs (Braun and Hildebrandt, 2017; Reiter and Leroux, 2017). This Research Topic spans multiple aspects of motile and primary cilia composition and function, ciliary mechanisms, and the physiopathological roles of cilia in different tissues. Here we describe some of the highlights.

## Cilia Biogenesis

Pioneering electron microscopy in the 1960's showed that mesenchymal cells assemble their cilia intracellularly, whereas polarized epithelial cells do so at the cell surface (Sorokin, 1962, 1968). While intracellular ciliogenesis mechanisms are relatively well-studied, those of the alternative cell surface route have remained enigmatic until recently, when work from the Alonso lab revealed a key role in the process for the cytokinetic midbody remnant. This lab now reviews the state of the art regarding how the midbody licenses membranes for ciliogenesis, and how it may have played a role in cilia evolution (Labat-de-Hoz et al.).

The actin cytoskeleton is a prominent player, not only in ciliogenesis regulation, but also in the control of cilia function, maintenance, length and disassembly, as reviewed by Smith et al. Although much remains to be learned, growing data suggest that actin polymerization, F-actin branching and stress fiber formation inhibit ciliogenesis, while actin depolymerization or depletion promotes it. However, this is contradicted in multiciliated cells, where apical F-actin stabilization is required to expand membrane surface, allowing the inter-spaced docking of many basal bodies. This review also updates the growing number of actin-binding cilia regulators, like LUZP1.

LUZP1 is a centrosomal ciliogenic regulator involved in actin filament bundling. Given the roles of cilia and actin in cancer, Bozal-Basterra et al. examined LUZP1's involvement in tumorigenesis. They find that LUZP1 loss in fibroblasts promotes cell migration, invasion, and apoptosis. LUZP1 also affected centriole numbers, nuclear size, and actin dynamics beyond F-actin bundling. These data suggest possible roles for LUZP1 in tumor progression and metastasis, underscoring how the interface between actin dynamics and ciliogenesis is fertile ground for the discovery of new therapeutic targets for both cancer and ciliopathies, as discussed by Smith et al.

Primary cilia modulate Wnt signaling output. However, how Wnt regulates ciliogenesis is less clear, as conflicting reports have described both positive and negative effects. To clarify this, Bernatik et al. looked at how Wnt pathway activation or suppression affect ciliogenesis and cilia length in several common cell lines. They find that Wnt inhibition does not impair ciliogenesis, while Wnt activation may moderately reduce it.

## Cilia Structure and Function

Regarding motile cilia, Jacinto et al. show that Pkd2 reduction in the ciliated cells from the zebrafish left-right organizer leads to 25% shorter cilia and decreased flow speed. Therefore, the authors alert about the inability to discern the role of Pkd2 in calcium signaling from its role in fluid mechanics during left-right development (Jacinto et al.). However, cilia motility may depend on non-ciliary proteins; mice lacking tight junction component Jam3 display phenotypes suggestive of motile cilia dysfunction (pulmonary dysfunction, hydrocephalus, and sperm defects). To test this, Mateos-Quiros et al. examined Jam3 in mouse airways. They found Jam3 labels apical sorting endosomes and cell-cell contacts in a subset of multiciliated cells. Although Jam3 is not required for ciliogenesis or ciliary motility, it affects transepithelial resistance, and its levels are differentiation and inflammation-dependent.

New insights about how contact between sperm and zona-pellucida (ZP) glycoproteins on the surface of the mouse oocyte cause activation of sperm and egg fertilization are provided by the groups of Balbach et al. This study revealed that sperm engagement with ZP proteins leads to opening of the sperm-specific, pH-sensitive Ca^2+^ conducting channel, CatSper. This process is apparently driven by an elevation of intracellular pH, which eventually leads to increased swimming and acrosomal exocytosis.

Cilia assembly and maintenance rely on bidirectional movement along the axoneme *via* intraflagellar transport (IFT) machinery. In Wang et al., authors review IFT roles in cilia assembly/disassembly, including ciliary transport of structural and membrane proteins, ectocytosis modulation, and how tubulin post-translational modifications (PTMs) influence IFT. The authors end by focusing on the ciliary length dynamics of renal epithelia.

One of the most abundant axonemal PTMs is glutamylation. Yang et al. review how this PTM modulates ciliary structure and function. They discuss how balanced function of distinct members of the tubulin tyrosine ligase-like (TTLL) family and of cytosolic carboxypeptidases (CCPs) is regulated at cilia. In primary cilia, altered glutamylation may affect axoneme architecture by modulating proteins such as the severing enzyme Spastin. Motile cilia beating is also affected by glutamylation. This PTM also regulates IFT dynamics, with expected implications for ciliary signaling and disease.

The ciliary membrane is continuous with the plasma membrane but exhibits a unique lipid composition, which is essential for correct ciliary function. Phosphoinositide lipids play key roles in cilia and ciliopathies, but their ciliary distribution had not been mapped at high resolution. Conduit et al. use STED superresolution microscopy to finely map phosphoinositides within the ciliary transition zone. They find that PIP_3_ and PI(4,5)P_2_ form two distinct and adjacent rings at the proximal transition zone membrane, with the PIP_3_ ring being slightly more distal. Consistently, phosphoinositide regulators (INPP5E) and effectors (phospho-AKT) also localize to these rings.

## Cilia in Specialized Cell Types

Cilia in different cell types have evolved to cope with different environmental challenges and signals. How this cilia diversity is related to the etiology of ciliopathies has been under investigation. In the retina, photoreceptors rely on a highly specialized and modified primary cilium, the outer segment (OS), which possesses an elaborate membrane system enriched in light-transducing molecules such as rhodopsin. The OS is highly dynamic, requiring daily renewal of 10% of its components. The structure and function of ciliated photoreceptors are reviewed by Barnes et al., who explore recent advances on the molecular players and mechanisms underlying OS biogenesis and maintenance. This work is complemented by that of Sánchez-Bellver et al., who review how syndromic and non-syndromic retinal ciliopathies are caused by mutations in over 100 ciliary genes affecting different aspects of ciliary photoreceptor trafficking.

Ma and Zhou highlight the critical roles of endothelial primary cilia in generating and maintaining vascular barriers. They review how these cilia fulfill these functions by regulating ciliary molecules like IFT components and polycystin 1 (PC1), and *via* signaling pathways like Hedgehog, Notch, and Wnt. In the blood vessel lumen, these cilia sense extracellular signals like blood flow, pH, calcium, and nitric oxide, and are required for early stages of angiogenesis. The correlation between endothelial cilia malfunction and vascular diseases makes endothelial cilia attractive therapeutic targets.

Also, skin cells grow primary cilia that regulate skin functions like immunity and hair growth. A comprehensive review by Toriyama and Ishii summarizes this understudied area highlighting the cellular and molecular mechanisms of the interplay between skin cilia and immune responses. They also explore the pathophysiological relevance of these processes, as well as the therapeutic potential of targeting skin primary cilia for atopic dermatitis among other skin diseases.

Primary cilia are important in neurodevelopmental disorders such as intellectual disability, autism spectrum disorders, and epilepsy. Hasenpusch-Theil and Theil reviewed how primary cilia play critical roles in neurogenesis, neuron migration, and neuronal circuit formation during corticogenesis. They provide evidence that, during cortical morphogenesis, primary cilia are required to suppress Hh signaling mainly by regulating GLI3 repressor levels. In radial glial cells and neural stem cells, cilia have different, time-dependent roles, probably by regulating signaling pathways including Hh, mTORC1, and others.

## Cilia and Disease

Kobayashi et al. studied the role of cilia in pancreatic ductal adenocarcinoma (PDAC), showing that CEP164 depletion enhances clonogenicity of Panc1 cells. They suggest that lack of cilia promotes PDAC cell proliferation. CEP164 colocalizes with the GLI2 transcription factor at the mother centriole, controlling its activation and thereby inducing Cyclin D-CDK6 expression. The authors suggest CEP164 is a potential therapeutic target for PDAC.

Finally, Álvarez-Satta et al. delved into the molecular bases of Alström syndrome (ALMS), a ciliopathy associated with retinal dystrophy, obesity, diabetes, cardiomyopathy, and widespread fibrosis. ALMS is caused by mutations in the ALMS1 protein, knockdown of which in RPE1 cells leads to the formation of longer and misshapen cilia. These cells also displayed impaired responsiveness to TGF-β, which could help explain some of the pathology of Alström patients, like the cardiac defects and fibrosis.

## Conclusions

The papers in this Research Topic present an integrated view of cilia function, ranging from ciliogenesis mechanisms, the sensory and motile functions of cilia in a variety of cell types, and their roles in both health and disease. The many insights gathered in this article series will help guide future advances in the field. In the next years, we expect important developments in our understanding of the detailed molecular structure of cilia in different cell types, leading to deeper mechanistic insight into structure-function relationships. This in turn will shed light into the myriad pathophysiological roles of cilia, with potential therapeutic implications.

## Author Contributions

HS and FRGG coordinated and finally organized the text. All authors contributed to writing and editing the Editorial.

## Funding

FRGG was funded by grants from the Spanish Ministry of Science and Innovation (PID2019-104941RB-I00) and from CIBERER (ACCI-2020) and HS was funded by grants of Fundação para a Ciência e a Tecnologia (FCT) Portugal (UIDB/00100/2020 and UIDP/00100/2020).

## Conflict of Interest

The authors declare that the research was conducted in the absence of any commercial or financial relationships that could be construed as a potential conflict of interest.

## Publisher's Note

All claims expressed in this article are solely those of the authors and do not necessarily represent those of their affiliated organizations, or those of the publisher, the editors and the reviewers. Any product that may be evaluated in this article, or claim that may be made by its manufacturer, is not guaranteed or endorsed by the publisher.

## References

[B1] Anvarian Z. Mykytyn K. Mukhopadhyay S. Pedersen L. B. Christensen S. T. (2019). Cellular signalling by primary cilia in development, organ function and disease. Nat. Rev. Nephrol. 15, 199–219. 10.1038/s41581-019-0116-9 30733609 PMC6426138

[B2] Braun D. A. Hildebrandt F. (2017). Ciliopathies. Cold Spring Harb. Perspect. Biol. 9:a028191. 10.1101/cshperspect.a028191 27793968 PMC5334254

[B3] Reiter J. F. Leroux M. R. (2017). Genes and molecular pathways underpinning ciliopathies. Nat. Rev. Mol. Cell Biol. 18, 533–547. 10.1038/nrm.2017.60 28698599 PMC5851292

[B4] Sorokin S. (1962). Centrioles and the formation of rudimentary cilia by fibroblasts and smooth muscle cells. J. Cell Biol. 15, 363–377. 10.1083/jcb.15.2.363 13978319 PMC2106144

[B5] Sorokin S. P. (1968). Reconstructions of centriole formation and ciliogenesis in mammalian lungs. J. Cell Sci. 3, 207–230. 5661997 10.1242/jcs.3.2.207

